# Scalp high-frequency oscillation rates are higher in younger children

**DOI:** 10.1093/braincomms/fcab052

**Published:** 2021-03-23

**Authors:** Dorottya Cserpan, Ece Boran, Santo Pietro Lo Biundo, Richard Rosch, Johannes Sarnthein, Georgia Ramantani

**Affiliations:** 1 Department of Neuropediatrics, University Children's Hospital Zurich, 8032 Zurich, Switzerland; 2 Department of Neurosurgery, University Hospital Zurich, 8006 Zurich, Switzerland; 3 University of Zurich, 8006 Zurich, Switzerland; 4 Klinisches Neurozentrum Zurich, University Hospital Zurich, 8006 Zurich, Switzerland; 5 Children’s Research Centre, University Children's Hospital Zurich, 8032 Zurich, Switzerland

**Keywords:** paediatric epilepsy, scalp EEG, high-frequency oscillations, reliability, biomarker

## Abstract

High-frequency oscillations in scalp EEG are promising non-invasive biomarkers of epileptogenicity. However, it is unclear how high-frequency oscillations are impacted by age in the paediatric population. We prospectively recorded whole-night scalp EEG in 30 children and adolescents with focal or generalized epilepsy. We used an automated and clinically validated high-frequency oscillation detector to determine ripple rates (80–250 Hz) in bipolar channels. Children < 7 years had higher high-frequency oscillation rates (*P *=* *0.021) when compared with older children. The median test−retest reliability of high-frequency oscillation rates reached 100% (iqr 50) for a data interval duration of 10 min. Scalp high-frequency oscillation frequency decreased with age (*r* = −0.558, *P *=* *0.002), whereas scalp high-frequency oscillation duration and amplitude were unaffected. The signal-to-noise ratio improved with age (*r* = 0.37, *P *=* *0.048), and the background ripple band activity decreased with age (*r* = −0.463, *P *=* *0.011). We characterize the relationship of scalp high-frequency oscillation features and age in paediatric patients. EEG intervals of ≥10 min duration are required for reliable measurements of high-frequency oscillation rates. This study is a further step towards establishing scalp high-frequency oscillations as a valid epileptogenicity biomarker in this vulnerable age group.

## Teaser

Cserpan et al. report the impact of age on high-frequency oscillations recorded in the scalp EEG of children with epilepsy. Children <7 years had higher rates of high-frequency oscillations when compared with older children. EEG intervals of ≥10 min were required for the reliable estimation of these rates.

## Graphical Abstract

**Graphical Abstract:**
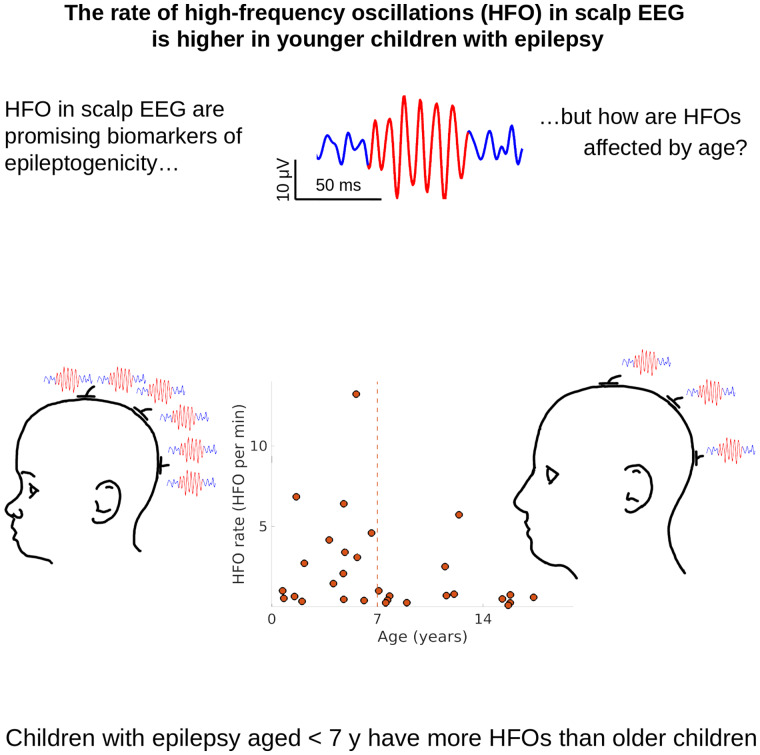

## Introduction

High-frequency oscillations (HFO), initially identified as epilepsy biomarkers in the intracranial EEG of patients undergoing presurgical evaluation for focal epilepsy, have recently also been recorded in scalp EEG, indicating their potential future use as non-invasive biomarkers in a broader population of patients with epilepsy.^1^^,^^2^ Beyond their use for delineating the epileptogenic zone and facilitating surgical decision making, HFO are currently investigated as potential biomarkers of epileptogenesis, disease severity and treatment response.^2^ Novel and reliable non-invasive EEG biomarkers are urgently required since other biomarkers currently in clinical use, such as spikes, lack specificity as they are sometimes recorded even in the absence of epilepsy.^3^^,^^4^ Furthermore, spike rates correlate with neither seizure frequency nor treatment response in patients with epilepsy.^5^ A novel biomarker of epileptogenesis that would guide timely treatment initiation, potentially even before seizure onset in selected patients, as well as efficiently monitor treatment response is poised to drastically change epilepsy management. Considering that epilepsy incidence is higher in early life,^6^ novel biomarkers are poised to have the largest impact in the paediatric age group, possibly improving outcome in this vulnerable age group. 

The utility of scalp HFO as a biomarker in paediatric epilepsy has been investigated in several recent studies that corroborated the correlation of scalp HFO with (i) disease severity in epileptic encephalopathies such as early infantile epileptic encephalopathy^7^ and West syndrome,^8^^,^^9^ self-limited focal epilepsies such as Rolandic epilepsy^10^ and its atypical forms^11^ and focal structural epilepsies^12^; (ii) development of CSWS (continuous sharp waves in sleep)^13–16^ and (iii) seizure risk at the presence of Rolandic spikes^17^ and tuberous sclerosis^18^ or after a first epileptic seizure, regardless of aetiology.^19^ All of these studies have included patients in a wide age range from early childhood to adolescence. Yet the impact of age itself on scalp HFO characteristics, potentially of crucial importance for further analysis, is still unclear.

The only indication of a possible age-related pattern in scalp HFO stems from a mixed cohort of children with (*n* = 6) and without epilepsy (*n* = 17).^20^ High HFO rates occurred in children under 5 years, whereas children over 13 years showed no HFO. However, this observation of decreasing HFO rates with age failed to reach statistical significance,^20^ in line with the lack of age-dependent changes in scalp HFO rates in a longitudinal paediatric tuberous sclerosis study.^9^ The observation of age-independent scalp HFO rates is surprising in light of the gradually increasing power in other higher frequency bands with age, as recently confirmed in a study of cortical rhythm maturation through childhood,^21^ thus adding to the controversy surrounding age-related changes in scalp HFO rates. Furthermore, although several studies have reported the frequency, duration and amplitude of pathological as well as physiological HFO in intracranial EEG,^22^^,^^23^ only a few studies have analysed these features in the scalp EEG,^9^^,^^20^ with only a single study to date having evaluated HFO changes with age, without finding evidence of a correlation.^9^ Finally, the modification of brain activity in various frequency bands during development^21^ may impact the signal-to-noise ratio (SNR) and thus the detectability of scalp HFO. There are considerable changes not only in brain physiology, but also in skull density and thickness, all of which may modify HFO signal characteristics, making it imperative to consider developmental HFO changes for the clinical implementation of HFOs as a reliable EEG biomarker in paediatric epilepsy.

In this study, we retrospectively analysed whole-night video-EEG in paediatric patients with epilepsy and implemented an automated HFO-detector to ensure a prospective, bias-free definition of clinically relevant HFO. We illustrate the changes occurring throughout childhood and adolescence on (i) scalp HFO rate and (ii) scalp HFO characteristics, including frequency, duration, amplitude and (iii) scalp HFO detectability and rate reliability. This precise delineation of age-dependent changes in HFO features will enhance diagnostic accuracy and facilitate the implementation of scalp HFO as an epilepsy biomarker in larger, less selected cohorts of paediatric epilepsy patients.

## Materials and methods

### Patient recruitment

We prospectively enrolled 30 consecutive children and adolescents (<18 years) with epilepsy that underwent whole-night video-EEGs at the University Children's Hospital Zurich, between January and September 2020. The clinical purpose of whole-night EEG included the differential diagnosis of epileptic versus non-epileptic events in patients with an established diagnosis of epilepsy (e.g. discriminating dystonic posturing from ictal events), determination of seizure and epilepsy syndrome classification, and pre-surgical evaluation. Epilepsy syndromes were classified as focal or generalized, and their aetiology as structural or genetic,^24^ depending on the electroclinical correlations, MRI findings and genetic results. Seizure frequency, as a measure of epilepsy severity, was given as the number of seizures per month based on long-term video-EEG for patients with daily seizures or on seizure diaries for patients with weekly, monthly or yearly seizures.

We selected one recording from each patient for further analysis that fulfilled the following inclusion criteria: (i) high sampling frequency (>1000 Hz), and (ii) containing at least 10 min of N3 sleep.

The collection of patient data and the scientific analysis were approved by and performed according to the guidelines and regulations of the local ethics committee (Kantonale Ethikkommission Zürich, KEK-ZH PB-2016–02055). All patients and their parents gave written informed consent before participating in the study.

### Scalp EEG recording and data selection

Whole-night video-EEG was recorded with 21 electrodes placed according to the international 10–20 system at a 1024 Hz sampling rate using the Micromed^®^ EEG recording system (Mogliano Veneto, Treviso, Italy). Impedances were typically ≤1 kΩ. Sleep stages were marked according to the American Academy of Sleep Medicine (AASM) criteria by experienced neurophysiologists. Continuous spike-and-wave during sleep (CSWS) was defined by a sharp wave index (SWI) exceeding 85% of NREM sleep.^25^

We selected NREM (specifically, N3) data intervals from the first 3 h of sleep, since pathological HFO are less contaminated by muscle artefacts, and their rates are higher in N3 sleep compared with other states^26^ and decrease with time spent in sleep. Scalp EEG intervals with visible artefacts and channels with continuous interference were excluded from further analysis. We divided the selected data into 5-min intervals for further processing, and only patients with more than two 5-min intervals were considered for further analysis. We considered the patients’ age in years as a continuous variable. For subgroup analysis, we divided the patients into a younger group (<7 years) and an older group (≥7 years).

### Automated HFO detection

We first re-referenced to a bipolar montage, using all combinations of neighbouring electrodes, which resulted in 52 bipolar channels. With this approach, we expected to capture the HFO activity more accurately. HFO detection was conducted with a clinically validated, automated HFO detector that has been described in detail earlier.^12^^,^^27–30^ In brief, the detector has three stages. Stage I determines a baseline amplitude threshold in time intervals with high Stockwell entropy (low oscillatory activity) in the ripple band (80–250 Hz). Events exceeding the threshold are marked as events of interest (EoI). In Stage II, we select all EoI that exhibit a high frequency peak isolated from low frequency activity in the time−frequency space. In Stage III we apply the following criteria that are specific to scalp EEG. We reject all EoI that have an amplitude ≥40 µV or SNR <4 or that occurs simultaneously in homologous channels of the two hemispheres; co-occurrence was defined as partial overlap in time. There was no further visual validation of the events, rendering the algorithm fully automated. We considered only events in the ripple band (80–250 Hz) for further analysis.

HFO detection and analysis were performed blinded to clinical data, and HFOs were not considered for clinical decisions. We then controlled for the clinical plausibility of scalp HFO by comparing the localization of the channel with the highest HFO rate with the localization of spikes in scalp EEG and focal lesions in MRI.

### HFO characterization

For each patient, we calculated the channel-wise HFO rate by dividing the number of the detected HFOs on a channel by the total duration of the recordings.

To characterize the HFOs, we calculated the means of HFO frequency, duration and amplitude for the channel with the highest HFO rates. We define HFO frequency as the frequency of the crest and trough waves of the HFO, HFO duration as the duration that the Hilbert envelope of the HFO remains above the detection threshold, and the HFO amplitude as the highest peak-to-peak amplitude of the HFO. We included patients with >3 HFOs in the morphology characterization.

The SNR for each patient is calculated as the average of the SNRs of the detected HFOs on the channel with the highest HFO rate. The SNR of an HFO is estimated as the signal power within the duration of the HFO divided by the signal power 0.5 s before and after the HFO. The ripple band activity (RBA) is estimated as the average of the root-mean-square values of the filtered signal in the 80–250 Hz band in the channel with the highest HFO rate.

### Statistics

We describe distributions by their median and the interquartile range (iqr). We used non-parametric statistics. For comparisons, we used the Wilcoxon rank sum and the Wilcoxon signed rank tests. To quantify correlations, we used Spearman’s rank correlation. Hypothesis tests were calculated with MATLAB^®^ 2020a.

We modelled the HFO rate using the automatic linear modelling function of SPSS^®^ version T26. In order to analyse the relationship between the HFO rate and four potential predictors, we created a simple linear model and introduced an intercept. Considering the limited number of data points, we used the HFO rate as dependent and we chose four independent variables that we considered most relevant. We selected the logarithm of seizure frequency, CSWS (yes, no), age (younger, older) and RBA as predictor variables. Statistical significance was established at *P* < 0.05.

### Data and code availability

The raw EEG data and the HFO markings are freely available at the OpenNeuro platform (https://openneuro.org/datasets/ds003555/versions/1.0.1).

The software for the detection of HFOs is freely available at the GitHub repository (https://github.com/ZurichNCH/Automatic-High-Frequency-Oscillation-Detector).

## Results

### Patients' characteristics

We included 30 patients (16 female) with focal or generalized epilepsy (Table 1). The median age at the recording was 6.4 years (range 0.7–17.4 years). Nine patients had a generalized epilepsy, 5 related to a known disease mutation; the remaining 21 had a focal epilepsy, 19 associated with a known structural brain lesion.

**Table:** Table 1 Patient characteristics and HFO properties. The test-retest reliability of the HFO area was calculated for recording intervals of 10 min. y: years, f: female, m: male, nr: number, FCD: focal cortical dysplasia, (m)MCD: (mild) malformations of cortical development, PMG: polymicrogyria, CSWS: continuous spikes and waves during sleep.

Patient	Age (years)	Sex	Epilepsy classification	Aetiology	Seizures per month	Data intervals	HFO events	HFO/ min	Frequency (Hz)	Duration (ms)	Amplitude (µV)	SNR	Ripple band activity (µV)	Channel with highest HFO rate	Test− retest reliability 10 min (%)
1	5.6	F	Focal: parietal—CSWS	Structural: bilateral (L > R) perinatal ischaemic lesions	150	6	397	13.2	126	36	7.7	9.4	1.3	P3-O1	100
2	1.6	F	Generalized	Genetic: DMN1 mutation	60	4	137	6.8	97	55	10.5	6.7	1.6	T6-P4	100
3	4.8	M	Focal: temporal—CSWS	Structural: (radiologic suspicion of) FCD	0.01	17	545	6.4	122	37	10.3	8.1	1.9	T6-P4	100
4	12.4	F	Generalized—CSWS	Genetic	0.01	3	86	5.7	85	49	15.0	10.0	1.5	T5-P3	–
5	6.6	M	Focal: temporal—CSWS	Structural: perinatal thalamic bleeding, hippocampal sclerosis	0.01	3	69	4.6	121	40	10.0	12.3	1.5	T3-F3	–
6	3.8	F	Focal: hemispheric	Structural: FCD 1a	15	5	104	4.2	121	55	10.0	9.1	1.6	F8-F4	100
7	4.9	M	Focal: temporal	Structural: MMCD	4410	9	153	3.4	118	48	7.6	9.4	1.2	P3-O1	100
8	5.7	F	Generalized	Genetic: KMT2E mutation	18000	5	77	3.1	109	54	11.9	14.8	1.3	T6-O2	100
9	2.2	M	Focal: hemispheric	Structural: FCD 1a	0.01	9	122	2.7	115	52	6.6	7.1	1.1	T5-C3	100
10	11.5	M	Focal: parietal	Structural: FCD 2a	2	17	213	2.5	106	45	9.6	14.9	1.5	F3-C3	100
11	4.7	F	Focal: frontal	Structural: MCD R frontal, corpus callosum dysgenesis	240	11	114	2.1	113	53	8.3	9.8	1.2	Pz-O2	60
12	4.1	M	Focal: hemispheric	Structural: Perinatal stroke	780	8	58	1.4	110	37	9.3	8.4	1.5	F8-F4	100
13	0.7	M	Generalized	Unknown: Watanabe syndrome	0.01	2	10	1.0	174	24	8.3	4.4	2.2	F7-T3	–
14	7.1	F	Generalized	Genetic: SYN1 mutation	60	3	15	1.0	107	35	9.5	8.2	1.7	F3-Cz	100
15	12.1	M	Focal: parietal	Structural: Tuberous sclerosis	90	3	12	0.8	87	51	7.9	7.0	1.2	P4-Cz	–
16	15.8	F	Generalized	Unknown: Lennox Gastaut syndrome	300	4	15	0.7	119	51	7.9	6.0	1.5	P4-Cz	0
17	11.6	F	Generalized	Unknown	1.5	9	32	0.7	97	43	15.3	13.6	1.3	F8-T4	100
18	7.8	F	Focal: temporal	Structural: glioma, corpus callosum hypoplasia, nodular perventricular heterotopia	90	10	34	0.7	120	85	13.5	8.6	1.1	Fp1-F3	100
19	1.5	F	Focal: frontal	Structural: (radiologic suspicion of) FCD	360	4	13	0.6	129	46	16.0	11.7	1.6	F7-T3	100
20	17.4	M	Focal: hemispheric	Structural: hemiconvulsion-hemiplegia epilepsy syndrome (HHE)	0.3	5	15	0.6	106	45	13.4	14.1	1.0	Fp1-Fp2	100
21	0.8	F	Generalized	Genetic: KCNA1 mutation	180	12	32	0.5	114	43	14.1	9.3	1.8	Fp1-Fp2	87
22	7.7	F	Focal: frontal	Unknown	270	5	13	0.5	109	43	15.9	12.4	1.3	Fp1-F3	100
23	15.3	F	Focal: frontal	Unknown	210	4	10	0.5	92	42	11.9	18.4	1.0	F7-T3	100
24	4.8	M	Focal: hemispheric	Structural: postnatal stroke—aneurysma rupture	0.01	13	31	0.5	137	30	6.6	6.2	1.2	P3-Pz	43
25	6.1	M	focal: Bilateral	Structural: intracerebral haemorrhage, periventricular leukomalacia	300	12	24	0.4	96	37	17.0	19.9	1.1	F3-C3	27
26	2.0	F	focal: Frontal	Structural: bilateral MCD with PMG/FCD L frontal, corpus callosum agenesis, bilateral periventricular nodular heterotopia	60	7	12	0.3	116	38	13.9	14.0	1.5	T3-F3	83
27	8.9	M	Focal: temporo-occipital	Structural: (radiological suspicion of) FCD	750	3	4	0.3	79	37	9.5	12.1	1.1	T5-O1	0
28	7.6	M	Focal: temporal	Structural: pilocytic astrocytoma	150	7	9	0.3	115	70	17.4	8.2	1.8	Fp2-F8	50
29	15.8	M	Focal: temporal	Structural: hippocampal sclerosis	0.17	7	9	0.3	93	39	13.8	17.2	1.3	Fp1-F3	17
30	15.7	F	Generalized	Unknown	4	4	2	0.1	149	30	21.0	4.3	2.0	Fp1-F3	0

The total duration of scalp EEG recordings that contained N3 data intervals from the first 3 h of sleep and was thus considered for further analysis was 1055 min with a median of 27.5 min per patient (range 10–85 min, iqr 25).

### HFO rates are higher in younger children

**Figure 1:**
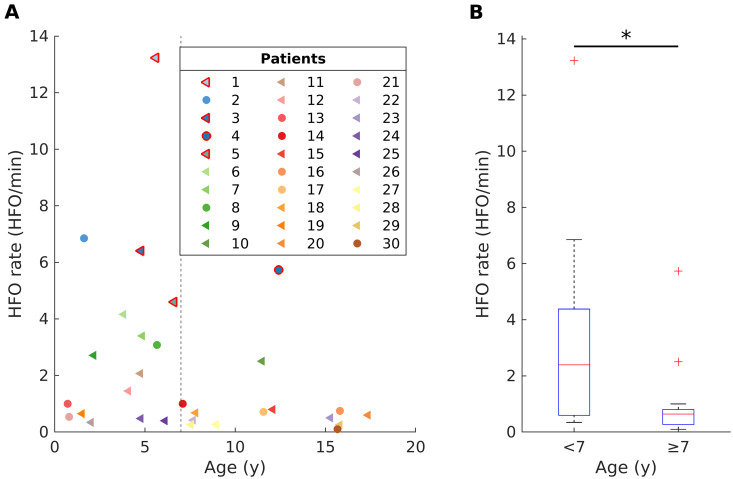
**The impact of age on scalp HFO rates in children and adolescents with epilepsy.** (**A**) Younger children (<7 years) showed higher scalp HFO rates than older children (≥7 years). Patients are numbered consecutively, as in Table 1, each depicted with a different colour. A triangle (*circle*) corresponds to a focal (*generalized*) epileptogenic zone. Markers with red edges denote the CSWS patients. (**B**) Scalp HFO rates in younger children (<7 years, *N* = 16, 2.4 HFO/min, iqr 3.8) were higher than in older children (≥7 years, *N* = 14, 0.6 HFO/min, iqr 0.5, Wilcoxon rank sum test, *P* = 0.021).

The median HFO rate across all patients was 0.77 HFO/min (iqr 2.6). Scalp HFO rates in younger children (<7 years, *N* = 16, 2.4 HFO/min, iqr 3.8) were higher than in older children (≥7 years, *N* = 14, 0.6 HFO/min, iqr 0.5, Wilcoxon rank sum test, *P *=* *0.021) (Fig. 1).

HFO rates did not differ significantly between focal (0.7 HFO/min, iqr 2.4) and generalized epilepsies (1.0 HFO/min, iqr 3.1) (Wilcoxon rank sum test, *P *=* *0.526). Similarly, HFO rates did not differ significantly between structural (0.8 HFO/min, iqr 2.8) and genetic epilepsies (3.1 HFO/min, iqr 5.1; Wilcoxon rank sum test, *P *=* *0.240). Patients with CSWS presented the highest HFO rates (6.1 HFO/min, iqr 4.7), regardless of aetiology.

Scalp HFO rates showed no significant correlation with seizure frequency in all patients (Spearman correlation, *P *>* *0.05) or in the subgroup of patients with focal structural epilepsy, after excluding those with CSWS and after hemispherotomy (Spearman correlation, *P *>* *0.05).

In the fitted linear model, the HFO rates were significantly lower in the older age group (≥7 years) and in the absence of CSWS but were not related to lower seizure frequency. The related coefficients, *t*-values, confidence intervals and significance values are presented in Table 2. The intercept gives the HFO rate when all independent variables are equal to 0. Therefore, in our case, the intercept gives the HFO rate estimate for CSWS in the younger age group with 0 RBA, and 1 seizure per month. It should be noted, however, that the intercept and its statistical significance are only theoretical values and have limited practical importance.

**Table 2:** Parameters of the linear model for HFO rate

Variable	Estimate	95% CI	*P*-value
CSWS (yes)	6.5	4.2 to 8.8	<0.001
Age group (>7 years versus ≤7 years)	−1.5	−2.9 to −0.1	0.037
Log(seizure frequency)	0.3	−0.1 to 0.7	0.171
RBA	−0.4	−2.8 to 2	0.749

HFO rate decreases in cases without CSWS and for those in the older age group.

### Reliability of HFO detection

Finally, we calculated the test**−**retest reliability of the HFO detection.

**Figure 2:**
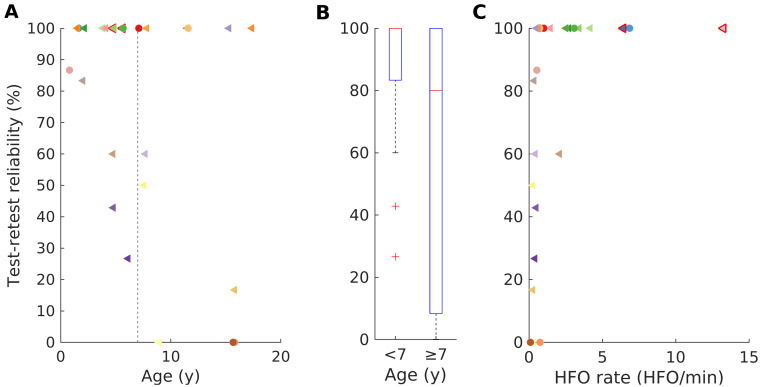
**Test−retest reliability of scalp HFO detection.** (**A**) The overall median reliability reached 100% (iqr 50%) with a 100% reliability being reached in 15/26 (58%) of children. (**B**) For older children (>7 years), the median reliability was 80% (iqr 92) but this value was not significantly different from younger children (Wilcoxon rank sum test, *P* = 0.208) due to the considerably wider range. (**C**) The reliability of scalp HFO detection increased with the HFO rate (*r* = 0.721, *P* < 0.001). For 15/26 patients (58%) the test**−**retest reliability reached 100%. Patients are marked as in Fig. 1.

We first calculated the reliability of 10-min data intervals (Fig. 2), including patients with a recording duration >20 min (*N* = 26). The overall median reliability reached 100% (iqr 50%) with a 100% reliability being reached in 15/26 (58%) of children. For younger children (<7 years) the median reliability also reached 100% (iqr 17%). For older children (>7 years), the median reliability was 80% (iqr 92) but this value was not significantly different from the younger children (Wilcoxon rank sum test, *P *=* *0.208) due to the considerably wider range.

We then calculated the reliability of 5-min data intervals, including all patients (*N* = 30). Compared with 10-min intervals, the median reliability dropped to 93% (iqr 59) in younger, and to 33% (iqr 47) in older children and there was a significant difference between age groups (Wilcoxon rank sum test, *P *=* *0.023). The reliability reached 100% only in 10 cases and was worse (Wilcoxon signed rank, *P *=* *0.004) than for the 10-min data intervals.

### HFO frequency decreases with age

**Figure 3:**
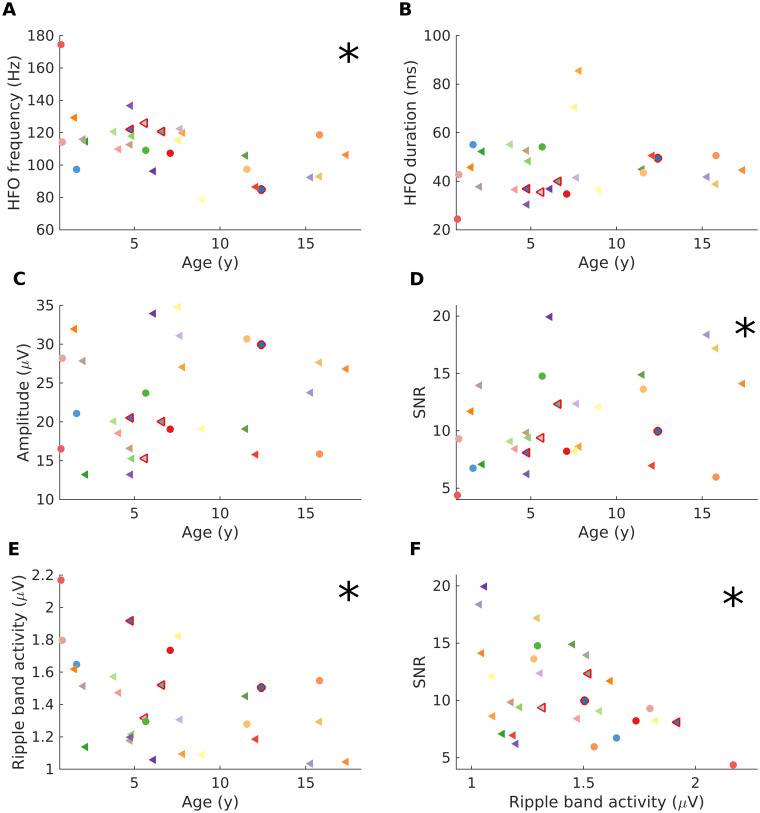
**The impact of age on scalp HFO characteristics.** (**A**) The scalp HFO frequency across patients decreased with age (*r* = −0.558, *P* = 0.002). (**B**, **C**) Neither the scalp HFO duration nor the scalp HFO amplitude correlated with age in our cohort. (**D**) The signal-to-noise rate (SNR) of scalp HFO improved with increasing age (*r* = 0.37, *P* = 0.048). (**E**) The RBA decreased with increasing age (*r* = −0.463, *P* = 0.011). (**F**) The SNR decreased with increasing RBA (*r* = −0.453, *P* = 0.014). Patients are marked as in Fig. 1. Stars mark significant correlations (Spearman’s *P* < 0.05).

The impact of age on HFO characteristics, including frequency, duration and amplitude is shown in Fig. 3. The division of our cohort into those younger and older than 7 years originates in the results of our exploratory analysis. Across patients, the median frequency was 112.6 Hz (iqr 22.8), the median duration 43.5 ms (iqr 14.1), and the median amplitude 20.5 µV (iqr 11.3). The scalp HFO frequency decreased with age (Spearman’s *r* = −0.558, *P *=* *0.002; Fig. 3A), whereas the HFO duration and the HFO amplitude showed no significant correlation with age (Fig. 3B).

### The SNR improves with age

The RBA and its influence on the SNR depended on age (Fig. 3). Across patients, the median SNR was 9.4 ± 3.9 (iqr 5.5) and the median RBA was 1.3 µV (iqr 0.4). The RBA decreased with increasing age (Spearman’s *r* = −0.463, *P *=* *0.011) (Fig. 3E). The SNR decreased with the increasing RBA (Spearman’s *r* = −0.453, *P *=* *0.014) (Fig. 3F). The SNR of scalp HFO improved with increasing age (Spearman’s *r* = 0.37, *P *=* *0.048) (Fig. 3D).

## Discussion

To our knowledge, we are the first to illustrate developmental changes in pathological scalp HFO rates and their characteristics (frequency, duration, amplitude and SNR). We provide evidence that scalp HFO rates decrease with age in paediatric epilepsy, independent of seizure frequency. We demonstrate that reliable measures of HFO detection can be achieved in 10-min data segments, with values being more consistent for younger children. We show that whilst scalp HFO frequency decreases with age, there is no age-related effect in HFO duration and amplitude. Finally, the SNR of scalp HFO improves with age in paediatric epilepsy, due to the decreasing background activity power. Our observations enable the decoding of scalp HFO signals, in a first step towards their implementation as a valid epilepsy biomarker in a clinical setting.

### HFO rates are higher in younger children

Scalp HFO rates were significantly higher in younger children with epilepsy in our study, in line with the significantly higher spike rates occurring in the younger age group.^31^^,^^32^ In fact, we may hypothesize that the same neuronal processes and conductivity changes determine the higher scalp HFO and spike rates in the first years of life. Moreover, our finding of decreasing HFO rates with age is in accordance with previous observations of much higher HFO rates in infants compared with adult patients^8^^,^^33^^,^^34^ and in preschool children compared with adolescents.^20^ This finding has obvious implications for scalp HFO detection, since scalp HFO as well as spikes, the standard non-invasive EEG biomarker, become rarer with advancing age, thus leading to a decrease in sensitivity over time. Interestingly, scalp HFO rates did not differ significantly depending on epilepsy classification, i.e. between focal versus generalized or structural versus genetic epilepsies. This extends previous observations from smaller, highly selected paediatric cohorts,^14^ underlining the generalizability of our findings in a wider population of children suffering from epilepsy beyond the subgroup with drug-resistant focal seizures. It should be, however, noted that, in our study, we have performed an exploratory analysis that needs to be confirmed in a new cohort, in whom the division at age 7 can be done a priori.

CSWS, irrespective of aetiology and seizure frequency (i.e. presence or absence of epileptic seizures), correlated with particularly high scalp HFO rates in our study. This observation is in line with previous work^13^^,^^15^^,^^16^^,^^35^ showing that epileptic encephalopathy with CSWS is associated with a considerably more abundant generation of HFOs compared with all other types of paediatric epilepsies. It should be noted that, in a recent study focussing on paediatric epilepsy with sleep-related spike activation, the highest HFO rates occurred in relation to younger age and presence of CSWS.^16^ This observation matches our findings of higher scalp HFO rates for younger children and those with CSWS. The abundance of pathological HFO in CSWS has been linked to the disruption of physiological brain networks at a sensitive time window for cognitive development and thus to developmental stagnation or regression.^13^^,^^16^

Scalp HFO rates showed no significant correlation with seizure frequency in our unselected cohort of children and adolescents with various epilepsy syndromes, aetiologies, and treatments. This finding stands in contrast to the significant positive correlation of scalp HFO rates with seizure frequency in a previous paediatric cohort with drug-resistant focal epilepsy that underwent serial scalp EEG recordings.^12^ This discrepancy may be attributed to the high heterogeneity of our current cohort, including patients with CSWS but only rare epileptic seizures as well as postsurgical recordings in patients with hemispheric disconnections. In addition, our current study considered only one scalp EEG recording from each patient. Longitudinal recordings as demonstrated in our previous work,^12^ may be better suited to evaluating such associations, particularly in highly heterogeneous patient groups.

### Reliability of scalp HFO detection

To confirm the reproducibility of our scalp HFO detection and establish the reliability of our findings, we performed a test**−**retest analysis, as previously developed by our group,^28^ investigating the spatial profile of HFO rates across several EEG intervals from each patient. In addition, to counteract HFO variability due to their occurrence according to the schedule of sleep homeostasis,^26^^,^^36^ we chose to analyse only data intervals from N3 sleep.

The reliability of scalp HFO detection was significantly higher for longer (10-min) than for shorter (5-min) EEG data intervals in our study, reaching a median of 100%. For shorter (5-min) data intervals, the reliability of scalp HFO detection was significantly higher for younger than for older children with epilepsy, since this measure is related to scalp HFO rates that are significantly higher in younger than in older children. In practical terms, while for younger children a reasonably high reliability is reached even when using only 5-min data intervals, for older children longer data segments may prove indispensable. Yet, it should be noted that the analysis of 10-min data intervals provides a considerably higher reliability compared with the analysis of shorter data intervals, even for the younger age group.

In this study, we only considered patients with at least 10-min data intervals for HFO detection and, based on our findings, we postulate that this approach should suffice for an accurate and consistent estimation of scalp HFO rates in most cases. We therefore recommend to record and analyze at least 10 min of N3 sleep to ensure a reliable scalp HFO detection in paediatric epilepsy,^37^ crucial for the implementation of this novel epilepsy biomarker in a clinical setting. HFO analysis requires stable spatial profiles over time that accurately reflect network properties, since data quality and representativity will determine the validity of results.^37^

### Scalp HFO characteristics change with age

The scalp HFO frequency decreased significantly with age in our study. We perceive this change within the context of brain maturation, as previously shown for the classical cortical rhythms of sleep EEG in early brain development.^21^^,^^32^^,^^38^^,^^39^

The duration and amplitude of scalp HFO remained unaffected by age in our study, in contrast to spikes that reportedly feature higher amplitudes and shorter duration in younger children.^32^ This finding is intriguing, since we would expect the changes in skull thickness and conductivity with advancing age^40^ to result in higher signal amplitude attenuation in older children.

Finally, the SNR of scalp HFO improves with age in paediatric epilepsy. This observation matches the properties of spikes that reportedly feature a higher SNR with advancing age due to the decreasing background activity power.^23^ Likewise, in our study, the improvement of SNR with age has been shown to inversely correlate with the decrease of RBA (Fig. 3F).

Quantitative measures for age-related changes in HFO characteristics are crucial for guiding HFO detection and classification both by the expert reviewer and by dedicated algorithms, since the HFO detector may require different thresholds for different age groups.

### Future directions

HFO detection in routine, non-invasive, scalp EEG recordings has introduced a novel and exciting study field, extending the range of patients with epilepsy that can profit from this approach beyond those with drug-resistant focal epilepsy undergoing invasive evaluation or epilepsy surgery.^12^ Non-invasive biomarkers for monitoring epileptogenesis, disease severity and treatment response, such as scalp EEG, are at the forefront of current research and appear particularly promising in the younger age groups. Considering the importance of timely intervention, particularly in early-onset epilepsy,^41–45^ non-invasively detected HFO could serve to determine epileptogenicity and thus seizure risk in relation to a focal lesion, guiding the timely initiation of therapeutic intervention. In addition, scalp HFO could serve to monitor therapy response, guiding anti-seizure drug tapering after successful drug treatment or epilepsy surgery. Scalp HFO can drastically improve epilepsy management in the first years of life and, thus, accomplish superior seizure and cognitive outcomes in this vulnerable population.

## Conclusion

We provide evidence that the scalp HFO rate decreases with age in paediatric epilepsy, independent of seizure frequency. We show that the reliability of HFO detection is high for 10 min EEG data intervals, suggesting 10 min as the minimum duration of sleep EEG recording for reliable HFO detection. Furthermore, we describe how the characteristics of HFO change throughout childhood and adolescence. This detailed insight improves the HFO interpretation. Our study is a further step towards establishing scalp HFO as a valid biomarker for epileptogenicity in the paediatric population.

## Abbreviations

AASM =: American Academy of Sleep Medicine
CSWS =: continuous spike-and-wave during sleep
FCD =: focal cortical dysplasia
HFO =: high-frequency oscillations
(m)MCD =: (mild) malformations of cortical development
NREM =: non-rapid eye movement sleep
PMG =: polymicrogyria
SNR =: signal-to-noise ratio
